# Probabilistic linking to enhance deterministic algorithms and reduce linkage errors in hospital administrative data

**DOI:** 10.14236/jhi.v24i2.891

**Published:** 2017-06-30

**Authors:** Gareth Hagger-Johnson, Katie Harron, Harvey Goldstein, Rob Aldridge, Ruth Gilbert

**Affiliations:** Administrative Data Research Centre England, University College London, London, UK, and Health and Social Care Information Centre, Leeds, UK; London School of Hygiene and Tropical Medicine, London, UK; Administrative Data Research Centre England, University College London, London, UK, and University of Bristol, Bristol, UK; University of Bristol, Bristol, UK, and Farr Institute, University College London, London, UK; Administrative Data Research Centre England, University College London, London, UK, University of Bristol, Bristol, UK, and Farr Institute, University College London, London, UK

**Keywords:** deterministic record linkage, evaluation, hospital discharge, probabilistic record linkage

## Abstract

**Background:**

The pseudonymisation algorithm used to link together episodes of care belonging to the same patient in England [Hospital Episode Statistics ID (HESID)] has never undergone any formal evaluation to determine the extent of data linkage error.

**Objective:**

To quantify improvements in linkage accuracy from adding probabilistic linkage to existing deterministic HESID algorithms.

**Methods:**

Inpatient admissions to National Health Service (NHS) hospitals in England (HES) over 17 years (1998 to 2015) for a sample of patients (born 13th or 28th of months in 1992/1998/2005/2012). We compared the existing deterministic algorithm with one that included an additional probabilistic step, in relation to a reference standard created using enhanced probabilistic matching with additional clinical and demographic information. Missed and false matches were quantified and the impact on estimates of hospital readmission within one year was determined.

**Results:**

HESID produced a high missed match rate, improving over time (8.6% in 1998 to 0.4% in 2015). Missed matches were more common for ethnic minorities, those living in areas of high socio-economic deprivation, foreign patients and those with ‘no fixed abode’. Estimates of the readmission rate were biased for several patient groups owing to missed matches, which were reduced for nearly all groups.

**Conclusion:**

Probabilistic linkage of HES reduced missed matches and bias in estimated readmission rates, with clear implications for commissioning, service evaluation and performance monitoring of hospitals. The existing algorithm should be modified to address data linkage error, and a retrospective update of the existing data would address existing linkage errors and their implications.

## Introduction

Data linkage algorithms are widely used to combine records that belong to the same individual. Errors in patient identifiers,^1^ data quality problems,^2^ missing data^3^ or imperfect linkage algorithms^1^ can produce two kinds of linkage errors: false matches, where two records belonging to different patients are linked (2) and missed matches, where two records belonging to the same patient are not linked. Linkage errors can bias the results of data analyses, with important implications for the accuracy of official statistics,^4^ and for data used for funding, planning or delivering services or for monitoring the relative performance of hospitals.

Bias due to linkage errors can artefactually alter differences between groups (for example, between hospitals, or age groups) by making differences bigger or smaller or changing the direction of the effect.^4^–^6^ The impact of bias due to linkage error can be compounded by low event rates or when sensitivity of an algorithm differs across cohorts.^7^ Analysts are rarely able to take linkage error into account in their analyses as linkage methods are rarely reported in detail,^8^ and few algorithms have been validated against good quality reference standard data sets.^1^,^6^ Hence, analysts using anonymised data, without identifiers, are often unaware of the extent of linkage error and cannot adjust for such error in their analyses.^9^

Data linkage errors can be addressed by improving both data quality and the algorithm used for linkage. Algorithms that use deterministic matching are popular, in part because they can be fully automated. However, deterministic algorithms designed to minimise false matches often have the disadvantage of a high missed match rate.^10^ The algorithm used to link together the records of care belonging to the same patient using National Health Service (NHS) hospitals in England [Hospital Episode Statistics (HES)], is thought to have a missed match rate of at least 4%.^1^ Although data quality has improved over time, the frequency with which key identifiers, such as NHS number, are missing disproportionately affects certain patient groups, leading to increased missed matched rates and hence to underestimates of readmission and mortality rates.^1^,^9^ HES is widely used for calculating costs, commissioning services, monitoring performance of NHS hospitals, evaluating services and monitoring health inequalities. Bias due to linkage error will affect all these analyses, and so has specific and important implications.

Probabilistic data linkage is known to produce more accurate linkage and less biased results^11^ than deterministic linkage, particularly in settings where data quality is poor.^12^ The aim of our evaluation was to determine if an additional probabilistic step to the existing deterministic algorithm used to link data on admissions to English hospitals (HES) would reduce the missed match rate and provide more accurate estimates of the relative risk of hospital readmission within one year, for different patient groups.

## Methods

### Population and databases

The HES administrative data set records care within English hospitals, from 1989/90 onwards.^13^ A deterministic linkage algorithm is used for internal data linkage, producing a pseudonym called the HES ID (HESID) that identifies the same patient when they are readmitted.^14^ Our study population comprised records where the date of birth was 13th or 28th of any month, appearing in the Admitted Patient Care data set from 1998 (the first available calendar year with data available on ethnic group and other relevant variables) to 2015 (the last available calendar year). These dates were chosen in order to avoid issues associated with transposition of days and months, and with commonly used default date values (1st and 15th).^15^ We restricted the sample to patients born in four years (1992, 1998, 2005, 2012), allowing us to consider both age and year of data collection. Analysis took place within the Health and Social Care Information Centre (HSCIC) in 2015 and 2016.

*Covariates* Age at admission was calculated using date of birth and admission date then grouped into 0–3, 4–7, 8–11, 12–15, 16–19 and 20–23. Sex was classified as male, female or missing. Ethnic groups were grouped into White, Mixed, Asian, Black, Chinese/Other and missing (missing included codes referring to unknown ethnic group). Postcode was used to identify records referring to foreign patients (which includes countries in the UK other than England), those with ‘no fixed abode’ (which includes homeless patients), and to calculate the index of multiple deprivation (IMD) 2004 score,^16^ a measure of socio-economic deprivation at a small area level. Five mutually exclusive socio-economic groups were created from postcode and IMD score: socio-economically deprived (most deprived quintile), not socio-economically deprived, missing postcode, foreign postcode and ‘no fixed abode’ postcode. For analyses after data linkage (described below) that considered patients and their risk of readmission, covariates may change over time, leading us to select the most commonly occurring category.

### Linkage procedures

Date of birth, sex, NHS number, local ID, provider code and postcode were used as the personal identifiers to match records.^14^ For the reference standard data set, ethnic group, general practitioner (GP) code, local authority code and the first three diagnostic codes (on the basis that 85% of records have up to three diagnostic codes) were used as additional identifying characteristics to ascertain true match status.^13^ Record linkage was performed in Microsoft SQL Server 2008, for deterministic and probabilistic matching.

#### Deterministic linkage

The existing deterministic algorithm operated by HSCIC to allocate HESID is not publicly available and is considered proprietary, but is described in sufficient detail elsewhere^14^ to be replicated using a range of programming languages. We wrote a version in SQL that has the same three steps: (1) Records are initially matched on the basis of partial or full agreement on date of birth, exact agreement on sex and exact agreement on NHS number; (2) Records are matched if partially agreed on date of birth, exactly agreed on sex, exact local ID within provider and exact postcode; (3) Records are matched if they agreed exactly on date of birth, sex and postcode. At this third step, communal postcodes are not considered and existing NHS numbers are disallowed. To match at step 3, NHS number and either local ID or provider code would have to be missing.^14^ Local ID within provider is a concatenation of provider and local ID, with zeros or spaces removed prior to linkage.^14^ Records with contradictory NHS numbers can be matched to the same HESID at step 2. Due to an ongoing error in compiling HES, most postcodes are missing for birth records prior to 2014. This technical issue means that all birth episodes extracted from hospitals into HES have blank postcodes, and therefore, limited geographic or socio-economic information is available. Only birth episodes incorrectly coded as another episode type (e.g. general episode) contain postcode.^17^ Allocation of NHS number at birth was introduced in 2005,^18^ generating linkage errors for multiple births before that time (given that NHS number was often missing and a match on local ID would not be allowed if postcode was missing).

#### Probabilistic linkage

We designed an additional probabilistic step to include unlinked records at step 3 because of missing NHS numbers or other identifiers. The probability that two identifiers would agree, given a match (*m* probability), was specified for each identifier: date of birth [0.95 (day), 0.94 (month), 0.91 (year)], 0.9 (sex), 0.9 (NHS number), 0.62 (local ID within provider) and 0.68 (postcode). These values were determined from preliminary analyses of the probabilities that NHS number agreed, and by evaluating their level of agreement in the reference standard data set. The probability that each identifier agreed, given a non-match (the *u* probability), was specified as 0.5 (sex), 0.03226 (day), 0.08333 (month), 0.05 (year), 0.00001 (NHS number), 0.00002 (local ID within hospital) and 0.00001 (postcode), respectively. Match weights for each identifier were calculated by dividing the *m* probability by the *u* probability and taking the log_2_ of the result.^19^ The total match weight for a record is the sum of the match weights for each identifier. Based on visual inspection of a histogram of match weights, we chose three thresholds above which a pair of records could be considered as an additional link: 10 (relaxed), 20 (middle) and 30 (strict). We then manually reviewed all scenarios producing additional links above each threshold, deciding on a final threshold of >21.5. This threshold was sufficiently relaxed to allow sex or date of birth to be missing or differ and to allow postcode to be missing if sex, date of birth and local ID agreed, but sufficiently strict to prevent additional false matches. Examples are available in Table 1 as part of the section on results.

#### Reference standard

A ‘reference standard’ HES data set was created by probabilistic matching using the same identifier that is used by the existing algorithm, in addition to a wider range of identifying characteristics (ethnic group, local authority, GP and diagnostic codes), and manual review. The *m* probabilities were based on the overall probabilities that identifiers agreed given a match on NHS number: local ID (0.8), postcode (0.7), ethnic group (0.8), local authority (0.9), GP (0.8) and agreement on one (0.3), two (0.1) or three (0.04) diagnostic codes. Following manual review, we found that false matches occurred primarily because of disagreement on NHS number and local ID, or because the record pairs may belong to multiple births. For these reasons, records were allowed to match in two scenarios: (1) total match weight >22.8 with the additional requirement that NHS number and local ID may not disagree; (2) NHS numbers were allowed to differ, if the level of agreement on other identifiers produced a total match weight >35 with the additional requirement that no multiple birth was indicated. Multiple births were defined as birth order or baby number >1, or ICD10 codes Z372 to Z377 inclusive. This decision was made on the basis of prior knowledge that NHS number can be wrong,^1^ but NHS number and local ID are the only two identifiers in this data set that can potentially distinguish multiple births sharing other identifiers.

### Ethical approval

As the analysis was a service evaluation to improve the quality of service provided by the HSCIC, which did not directly involve participants in research, we did not require NHS Research Ethics Committee ethical approval.^20^ The first author conducted all analyses internally at the HSCIC on record-level data, tables of results were shared with co-authors, and small cell sizes were suppressed to minimize the risk of disclosure. The study design and results were shared with HSCIC staff at three meetings between January and May 2016.

### statistical analysis

Before data linkage, we cleaned the data sets using existing data cleaning rules and data dictionaries.^13^ The quality of the data set was evaluated in terms of the proportion of missing data for different identifiers and different patient groups. After data linkage, we evaluated the missed match rate (at the record level), comparing the deterministic and probabilistic algorithms against the reference standard for all records within the entire study period (1998–2015). Sensitivity and specificity were calculated according to the standard formulae.^21^ The missed match rate is 1-sensitivity. To evaluate the impact of data linkage error on results (at the patient level), we modelled the risk of hospital readmission for patients within one year (the first admission linked to a second admission). Results from the deterministically linked and probabilistically linked data were compared to the reference standard. The percentage bias was estimated by comparing the coefficients (log odds) in logistic regression models with the coefficient in the model using the reference standard (the difference between the log odds of readmission in the comparison model and the reference standard, as a proportion of the log odds of readmission in the reference standard). In sensitivity analyses, we repeated results, comparing relaxed, middle and strict thresholds for probabilistic matching, to determine the impact of the choice on biased estimates of readmission. We also repeated analyses allowing the *m* probabilities to vary across three periods of data collection (1998–2003, 2004–2009, 2010–2015).

#### Patient involvement

There was no patient involvement in this service evaluation.

## Results

There were 418,046 records extracted from HES (calendar years 1998 to 2015). We removed 451 records where the year of admission was outside this range and 336 with no admission date available. Table 2 evaluates data quality for all records in the remaining extract of 417,259 records. Sex and local ID within hospital were very rarely missing (<0.1%) and are not shown. There was improvement in data quality over time. The number of records with missing NHS number fell from 43.8% (birth year 1992) to 0.7% (birth year 2012). The proportion of records with missing NHS numbers in the 1992 birth cohort is higher, because birth episodes were not captured by our sampling frame for this birth year. Postcode is missing for many birth records (prior to 2014) due to a system error,^17^ explaining the high proportion of missing postcodes in the 2005 (30.6%) and 2012 (47.3%) birth cohorts in our evaluation population. Postcode would usually be available for admissions after birth or where birth episodes had been incorrectly recorded as another type of episode. This is also shown in Table 4 that shows data quality across three data periods (1998–2003, 2004–2009 and 2010–2015) and additionally for different age groups. Table 2 shows that NHS number is more likely to be missing for ethnic minorities, foreign patients, those with no fixed abode and where the record has missing data in other fields (e.g. sex, ethnic group or postcode are also missing). Postcode is more likely to be missing when other fields are missing, particularly ethnic group, and is often missing for birth records prior to 2014. This highlights the potential for the rate of data linkage errors to vary across patient groups and produce biased results, given the strong emphasis placed on NHS number and postcode in the deterministic algorithm.

Linking records across the study period (1998–2015), the existing deterministic HESID algorithm has a missed match rate of 2.3% [95% Confidence Interval (CI) 2.2%, 2.4%] overall, but Table 3 shows that this was higher in older data years: from 1998 to 2003, this was 8.6% (95% CI 8.4%, 8.8%). There was variation across patient groups, with higher rates seen in ethnic minorities, foreign patients, those with no fixed abode and young infants. Specificity also improved over time, but even after the introduction of NHS number for babies in 2005 (which would reduce false matches generated by multiple births) the false match rate was higher than previously estimated (0.5% vs. 0.2%(1)). Table 3 shows that the additional probabilistic match step lowered the missed match rate for all patient groups.

### Causes of data linkage error

Table 1 shows the scenarios that would allow additional links not permitted by the existing algorithm. For example, in the first row, if NHS number, date of birth, local ID and postcode agreed but sex disagreed (as happened for 5 records), this would receive a match weight of 62.02 that would be permitted by our probabilistic algorithm but not by the existing deterministic algorithm. The most common scenario for missed matches was when NHS number was missing, local ID differed but sex, date of birth and postcode agreed (n = 3,642). This would not be permitted at step 3 of the existing algorithm because NHS number and local ID would have to be blank.^14^ Our reference standard considered these to be links, on the basis of other identifiers and identifying characteristics agreeing. The second most common scenario was for sex, date of birth and local ID to agree but postcode to be missing. This is not currently permitted but identified 1,809 additional links. An additional 722 links were identified where sex, date of birth and local ID agreed but postcode disagreed (Table 1).

### Impact on results (readmission rates for patients)

Whereas the missed matches in Table 3 refer to data linkage across the evaluation period for records, Table 4 considers the next aim of our evaluation – to evaluate the impact on the relative risk of hospital readmission for each patient within one year, comparing the existing deterministic algorithm (readmission rate 18.4%) with the additional probabilistic step (readmission rate 18.7%), adjusting for covariates. By comparing the coefficients with the same model run on the reference standard data (readmission rate 18.7%), we calculated bias - defined as the percentage by which the coefficient is under- or over-estimated. The number of patients decreases in the probabilistic model and the reference standard model, because fewer HESIDs are assigned to the same number of records (181,395 patients in the deterministic model, 176,990 with the additional probabilistic step, 175,773 in the reference standard).

Table 4 shows evidence of bias for nearly all patient groups, particularly males (6%), young infants (13%), children aged 8 to 11 (119%), young adults aged 16 to 19 (77%) or 20 to 23 (50%), Black (13%) and Chinese/Other (−3%) ethnic minority groups, patients living in areas of high socio-economic deprivation (9%), those with ‘no fixed abode’ (−70%) and in newer data years (−7%). The probabilistic match step reduced bias for nearly all patient groups, with the exception of foreign patients where it increased from 2% to 14%, although this involved a small number of patients (n = 142).

In sensitivity analyses (Table 5), relaxing the threshold for the additional probabilistic step lowered the missed match rate further, particularly for older data years, but increased the false match rate. A stricter threshold lowered the false match rate but increased the missed match rate.

## Discussion

Our results show missed matches that are produced by an existing deterministic algorithm that is used to link together hospital records in England within HES (inpatients) and the most common scenarios that create these data linkage errors. An additional probabilistic step reduced the number of missed matches, particularly for common scenarios where local ID agreed but other identifiers such as postcode were missing. Analyses of data that were linked using the additional probabilistic step had less biased estimates of hospital readmission rates for certain patient groups (e.g. ethnic minorities). Although the mismatch rate improved in recent years, there were discernible improvements in mismatch rates in virtually all patient groups and throughout the 17 years of analysis. The technique is particularly well suited to this administrative data source, where data quality is poor (particularly in older data years) but the implications of missed matches are serious – given that the HES data are widely used for commissioning and research. The reference standard we created additionally shows that other identifying characteristics (ethnic group, local authority, GP and diagnostic codes) can be used to substantially improve linkage success.

The strength of our evaluation is that it is the first attempt to evaluate data linkage error between multiple episodes of care for patients within the HES longitudinal data set. We previously showed that applying the HESID algorithm to link multiple episodes of paediatric intensive care data produced a false match rate of 0.2% and a missed match rate of >4%.^1^ In this study, the missed match rate was 2.3% overall but ranged from 8.6% (1998–2003) to 0.4% (2010–2015), with marked variation across patient groups.

A second strength of our evaluation was that we quantified the mechanisms that caused data linkage errors. A relatively small number of common scenarios created missed matches (Table 1). This has important implications for HES because it shows that the current deterministic algorithm is too strict, preventing matches that are very likely to be correct (e.g. sex, date of birth and local ID agree but postcode is missing; sex is missing but other identifiers agree; NHS number may be incorrect but other identifiers agree). The deterministic algorithm could be improved with additional deterministic steps that address these specific scenarios, or an additional probabilistic step could be introduced that automatically allows all scenarios above a threshold. Probabilistic matching is suitable for data sets where only one or two identifiers might have problems,^3^ because it can evaluate the overall level of agreement across all identifiers. It additionally allows situations in which NHS number might be valid, but incorrect.^1^ The technique was particularly useful for highlighting the benefit of local ID within hospitals, not currently allowed unless postcode also agrees. A relatively small number of additional links were captured by probabilistic matching, but small improvements in linkage error benefit certain subgroups (e.g. infants, young adults, ethnic minorities, foreign patients, those with ‘no fixed abode’ and those with poor quality data).

A limitation of our approach is that we cannot determine whether additional links are correct in relation to an external reference standard data set, since none exists for HES. Our analysis can be further extended using a recently developed method^22^ that uses all possible matches and their weights, rather than taking only those above a fixed threshold, but we have not pursued this further here. It may also be possible to improve linkage error by allowing *m* and *u* probabilities to change depending on the frequencies of different values for identifiers, which we did not consider here.^23^ The rate of change in postcodes, for example, will differ for different age groups,^24^ and the probability that NHS number or local ID agrees for a match may increase over successive data years. In our reference standard data set, we considered exact matching on up to three diagnostic codes, but future evaluations could consider clusters of disease codes that are likely to be more stable over time.^25^ A major limitation was that we focused on records for children and adolescents, meaning that results may not generalise to records for adults. Many of the mechanisms generating linkage error will, however, be similar across the age range, and the methods we propose can be used in other data sets.

Given that Accident and Emergency data is known to be lower quality than inpatient records, our results represent a ‘best case’ scenario in terms of linkage error for hospital data in England as a whole. In Accident & Emergency settings, there may be less opportunity to check patient identifiers and the proportion of missing data is higher.^9^,^26^ It is also likely to be worse when additionally considering records where date of birth is missing, incorrect or estimated with a ‘default’ date – our sampling frame was created using date of birth assumed to be valid and correct. These scenarios were excluded from our evaluation but could be addressed by probabilistic matching that would allow these records to link if agreement on other identifiers was sufficiently high. Although the probability that two identifiers agree for a match may change in different data sets, the threshold can be adjusted so that probabilistic matching is useful even for lower quality data sets.

The evaluation extends previous studies of apparent false matches in pseudonymised HES extracts^9^ and a preliminary estimate of the false and missed match rate when applying the HESID algorithm to a well-curated clinical data set.^1^ For the first time, the patient identifiers in HES (and additional identifying characteristics) were used to create a reference standard that could be used to evaluate the existing deterministic algorithm and identify which scenarios generated data linkage errors. The results show that there are vulnerable patient groups who are disadvantaged by the current algorithm, such as those without NHS numbers. Patients with ‘no fixed abode’ include the homeless, who have important healthcare needs and are frequently readmitted.^27^ Without an NHS number or postcode, their records are difficult to link, but probabilistic linkage can help if a local ID is available at the hospital. Our results will be particularly important for evaluating the health outcomes of vulnerable and mobile populations who are less likely to have NHS numbers.

### Implications for research

Future evaluations need to consider whether different match weights and threshold are needed for different hospitals. The accuracy of local ID for some hospitals may not be the same as for others, and we have previously shown that there is significant variation in data linkage error across hospitals in England.^9^ Further evaluations are necessary that determine how good local ID is in each hospital, at correctly identifying patients, particularly when NHS number is missing. Most patients in our study population will have a birth record that will increase the prevalence of blank postcodes relative to those whose birth was not recorded in HES. Evaluations of older adults and the elderly would be useful, and an evaluation of the impact of linkage error on mortality estimates. Although we considered a long time window for linking records, we considered readmissions within one year for patients. Over long periods, there is more opportunity for linkage error. There is a clear need for a reference standard data set that can be used to check patient identifiers for several administrative health data sets.

### Implications for practice

Even in recent years, the existing HES algorithm generates mismatch rates in some groups that result in clinically important biases in estimated readmission rates, thereby underestimating service use, health needs and comorbidity. Mismatch rates are likely to similarly underestimate mortality rates.^28^ Improvements to the algorithm for future years should be accompanied by retrospective linkage to update existing HESIDs. This is particularly important for infants who did not automatically acquire NHS numbers at birth prior to 2005, and whose birth episodes did not contain a postcode before 2014. Interpreting trends over time in readmission rates is problematic if these partly reflect improvements in data linkage. Also for infants, it is very important to correctly link a patient to a birth episode and maternity episode so that critical birth characteristics can be linked into children’s health care trajectories. HES is widely used for commissioning and research and it is imperative to address data quality issues. HES is also linked to external data sets that can further introduce problems if the internal linkage problems are not addressed.

## Conclusion

Deterministic linkage of hospital administrative data is prone to generate missed matches, which produces biased estimates of hospital readmission for vulnerable patient groups and for older data. Probabilistic data linkage is suitable for data sets like HES where data quality is poor, and it can highlight the benefits of making better use of particular identifiers such as local patient ID within hospitals. The algorithm can be changed to improve future record linkage, but a retrospective update is also required to address linkage error in existing data. It is important to evaluate and address linkage error and data quality,^29^ particularly for this data set that is used to allocate >£100 billion of public resources annually, and to plan and deliver health services. Development of an external, reference or ‘gold’ standard data set that could identify patients across a range of data sets, even where NHS number was not available, would be extremely useful.

## Figures and Tables

**Table 1:** Scenarios that resulted in unlinked records using deterministic linkage were subsequently linked following probabilistic linkage, ranked from most likely to be correct to least likely

NHS number	Sex	Date of birth	Local ID within hospital	Postcode	Match weight	n
A	D	A	A	A	62.02	5
A	.	A	A	A	62.02	55
A	D	A	A	.	45.96	10
A	.	A	A	.	45.96	5
A	A	A	D	A	44.49	9
A	D	A	A	D	44.32	5
A	.	A	.	A	42.75	22
.	.	A	A	A	42.24	25
.	D	A	A	A	42.24	14
A	.	A	D	A	41.32	41
A	D	A	D	A	41.32	9
.	A	A	A	.	29.35	1809
A	A	A	D	.	28.43	7
.	A	A	A	D	27.71	722
A	A	A	D	D	26.79	76
.	.	A	A	.	26.18	2
.	D	A	A	.	26.18	3
.	A	A	.	A	26.14	5
D	A	A	A	.	26.03	8
A	.	A	D	.	25.26	2
A	D	A	D	.	25.26	9
.	A	A	D	A	24.71	3642
.	.	A	A	D	24.54	2
.	D	A	A	D	24.54	10
D	A	A	A	D	24.39	5
A	.	A	D	D	23.62	5
A	D	A	D	D	23.62	11
.	.	A	.	A	22.97	1

*Note*. A = identifier agreed; D = identifier disagreed; . = identifier missing.

**Table 2:** Number (%) of records with missing nhs number or postcode by birth year (inpatient hospital episodes from 1998 to 2015)

		Birth year (for records with day of birth 13th and 28th of each month in these years)										

		1992 (n = 100,443)			1998 (n = 120,470)			2005 (n = 106,450)			2012 (n = 89,896)		

Record characteristics		NHS number (%)	Postcode (%)		NHS number (%)	Postcode (%)		NHS number (%)	Postcode (%)		NHS number (%)	Postcode (%)
Overall	n	43.8	3.8	n	4.6	12.3	n	5.3	30.7	n	0.7	47.3
*Sex*												
Missing	138	15.9	1.4	166	66.3	16.9	27	55.6	33.3	19	10.5	57.9
Male	60,285	4.2	3.8	54,779	37.6	13.0	47,939	5.5	33.0	40,849	0.7	50.4
Female	40,020	7.7	3.9	65,525	35.6	11.7	58,484	4.9	28.7	49,028	0.7	44.7
*Ethnic group*												
Missing	18,988	11.3	3.9	48,098	53.5	16.9	19,938	8.5	47.5	7,966	2.1	40.6
White	70,455	3.7	4.0	59,977	24.0	9.3	67,887	4.2	26.1	62,037	0.5	47.6
Mixed	1,264	4.4	4.0	1,023	4.7	2.1	2,755	4.3	26.7	3,876	0.6	51.6
Asian	4,643	7.8	2.6	5,529	25.8	7.2	8,473	4.1	28.6	9,027	0.6	49.4
Black	3,103	6.9	2.2	2,909	33.2	6.6	4,665	6.4	32.0	3,963	1.2	50.0
Chinese/Other	1,990	14.2	2.7	2,934	49.6	16.1	2,732	7.4	29.3	3,027	1.6	44.1
*Socio-economic group*												
Missing	3,836	8.2		14,784	61.7		32,644	6.5		42,523	0.4	
Low deprivation	66,956	5.1		75,884	32.3		51,755	4.1		33,525	0.8	
High deprivation	28,921	5.3		29,438	34.3		21,613	4.6		13,601	0.9	
Foreign	620	57.6		341	80.6		324	78.7		214	43.5	
No fixed abode	110	21.8		23	39.1		114	14.0		33	0.0	
*Episode type*												
Other episode				83,356	17.1	2.8	66,454	4.5	2.7	45,200	1.1	2.5
Birth episode				37,114	80.1	32.1	39,996	6.3	77.0	44,696	0.3	92.6

*Note*. Missing data on postcode refer to missing after excluding invalid or communal postcodes, and postcodes denoting ‘no fixed abode’ or foreign patients. The ‘mixed’ ethnic group was not recorded until April 2001 but appears in the 1992 and 1998 cohorts if taken from episodes from 2001 onwards. Proportions of missing data for local patient ID within provider are very small (<0.1%) and not shown here. High deprivation is defined as the most deprived quintile of IMD2004

**Table 3:** Percentage (95% CIs) of records classified as missed matches compared with reference standard following deterministic and probabilistic data linkage

	Records from 1998 to 2003 (n = 99,220)		Records from 2004 to 2009 (n = 128,666)		Records from 2010 to 2015 (n = 189,373)	

	Deterministic	Probabilistic	Deterministic	Probabilistic	Deterministic	Probabilistic
Links	58,768	62,941	100,282	101,086	153,226	153,547
Specificity %	0.985	0.981	0.987	0.996	0.998	0.997
Sensitivity %	0.914	0.976	0.993	0.994	0.997	0.999
Missed match %	8.6 (8.4, 8.8)	2.4 (2.2, 2.5)	1.3 (1.2, 1.3)	0.4 (0.4, 0.5)	0.4 (0.3, 0.4)	0.1 (0.1, 0.1)
*Age*						
0–3	12.1 (11.8, 12.5)	3.2 (3.1, 3.4)	1.4 (1.3, 1.4)	0.4 (0.3, 0.4)	0.2 (0.2, 0.3)	0.0 (0.0, 0.1)
4–7	3.8 (3.4, 4.1)	1.3 (1.1, 1.5)	1.7 (1.5, 2.0)	0.8 (0.6, 1.0)	0.4 (0.3, 0.5)	0.2 (0.1, 0.2)
8–11	2.2 (1.9, 2.4)	0.6 (0.4, 0.7)	1.7 (1.5, 2.0)	0.7 (0.5, 0.9)	0.3 (0.2, 0.4)	0.1 (0.0, 0.1)
12–15			0.7 (0.5, 0.8)	0.2 (0.1, 0.3)	0.8 (0.7, 1.0)	0.5 (0.4, 0.6)
16–19			0.6 (0.5, 0.7)	0.1 (0.0, 0.2)	0.6 (0.5, 0.7)	0.3 (0.2, 0.3)
20–23					0.2 (0.2, 0.3)	0.1 (0.0, 0.1)
*Sex*						
Missing	100.0 (all missed)	21.0 (14.6, 26.5)	100.0 (all missed)	40.0 (9.6, 58.3)	100.0 (all missed)	16.7 (1.8, 21.9)
Male	8.6 (8.3, 9.0)	2.4 (2.3, 2.6)	1.3 (1.2, 1.4)	0.4 (0.3, 0.5)	0.3 (0.3, 0.3)	0.1 (0.1, 0.1)
Female	8.2 (7.9, 8.5)	2.2 (2.1, 2.4)	1.3 (1.2, 1.4)	0.4 (0.4, 0.5)	0.4 (0.3, 0.4)	0.1 (0.1, 0.2)
*Ethnic group*						
Missing	10.4 (10.1, 10.8)	2.7 (2.6, 2.9)	2.2 (2.0, 2.4)	0.6 (0.5, 0.7)	0.9 (0.7, 1.0)	0.2 (0.1, 0.3)
White	6.8 (6.5, 7.1)	2.0 (1.8, 2.1)	1.0 (0.9, 1.0)	0.4 (0.3, 0.4)	0.3 (0.2, 0.3)	0.1 (0.1, 0.1)
Mixed	3.7 (0.1, 4.4)	0.9 (-0.9, 1.8)	1.0 (0.6, 1.3)	0.3 (0.1, 0.4)	0.2 (0.1, 0.3)	0.2 (0.1, 0.3)
Asian	7.0 (6.0, 8.0)	2.2 (1.6, 2.6)	1.0 (0.8, 1.2)	0.3 (0.2, 0.4)	0.6 (0.4, 0.7)	0.2 (0.1, 0.2)
Black	7.0 (5.5, 8.3)	2.3 (1.4, 2.9)	1.4 (1.0, 1.7)	0.4 (0.2, 0.6)	0.4 (0.2, 0.5)	0.1 (0.0, 0.2)
Chinese/Other	11.7 (10.1, 13.2)	2.7 (1.8, 3.3)	4.4 (3.5, 5.3)	0.6 (0.3, 0.9)	0.9 (0.6, 1.1)	0.1 (0.0, 0.2)
*Socio-economic group*						
Missing	26.4 (25.2, 27.6)	4.3 (3.8, 4.9)	1.7 (1.5, 1.8)	0.5 (0.4, 0.6)	0.2 (0.1, 0.2)	0.1 (0.0, 0.1)
Low deprivation	7.1 (6.8, 7.3)	2.2 (2.1, 2.4)	1.0 (0.9, 1.1)	0.4 (0.3, 0.4)	0.3 (0.2, 0.3)	0.1 (0.1, 0.1)
High deprivation	6.8 (6.4, 7.1)	2.2 (2.0, 2.4)	1.1 (1.0, 1.3)	0.5 (0.4, 0.5)	0.3 (0.2, 0.3)	0.2 (0.1, 0.2)
Foreign	69.4 (61.2, 78.1)		66.3 (59.8, 73.1)	1.5 (-0.2, 3.2)	31.6 (27.7, 35.4)	0.8 (0.0, 0.9)
No fixed abode			8.7 (2.0, 12.0)	0.0 (none missed)		

*Note*. Number of records in each category shown in Table 6

**Table 4:** Variation in odds ratios (95% cIs) and percentage bias for demographic risk factors for hospital readmission within one year according to hesId, comparing data linkage algorithms

	Reference standard (n = 175,773)			Deterministic (n = 181,395)			Det+Probabilistic (n = 176,990)	

		Odds ratio (95% CI)		Odds ratio (95% CI)	Bias^a^		Odds ratio (95% CI)	Bias^a^
1-year readmission rate	n	18.7%	n	18.4%		n	18.7%	
*Total*	175,773		181,395			176,990		
Male^b^	85,887	1.08 (1.05, 1.10)	88,243	1.07 (1.04, 1.10)	6%	90,303	1.07 (1.05, 1.10)	2%
Female^b^	89,710	(reference)	92,818	(reference)		86,484	(reference)	
*Age group*								
0 to 3	119,742	3.39 (3.22, 3.58)	123,712	2.88 (2.74, 3.02)	13%	120,186	3.40 (3.23, 3.58)	0%
4 to 7	14,856	(reference)	15,510	(reference)		15,137	(reference)	
8 to 11	10,787	1.08 (1.00, 1.16)	11,306	0.99 (0.92, 1.06)	119%	10,945	1.07 (0.99, 1.15)	15%
12 to 15	8,920	0.94 (0.87, 1.02)	9,186	0.83 (0.77, 0.89)		9,049	0.93 (0.86, 1.00)	
16 to 19	10,080	1.19 (1.11, 1.28)	10,239	1.04 (0.97, 1.12)	77%	10,243	1.18 (1.10, 1.27)	6%
20 to 23	11,388	1.31 (1.23, 1.41)	11,442	1.15 (1.07, 1.23)	50%	11,430	1.31 (1.23, 1.41)	1%
*Ethnic group*								
Missing	44,733	0.52 (0.50, 0.54)	47,518	0.51 (0.50, 0.54)	−1%	45,044	0.52 (0.51, 0.54)	1%
White	103,831	(reference)	106,070	(reference)		104,603	(reference)	
Mixed	3,978	0.96 (0.88, 1.05)	4,005	0.98 (0.90, 1.07)		3,989	0.97 (0.88, 1.05)	
Asian	11,481	0.98 (0.93, 1.04)	11,731	0.98 (0.93, 1.04)		11,543	0.99 (0.94, 1.04)	
Black	6,457	0.78 (0.73, 0.84)	6,557	0.81 (0.75, 0.87)	13%	6,491	0.8 (0.75, 0.86)	9%
Chinese/Other	5,293	0.73 (0.67, 0.79)	5,514	0.72 (0.67, 0.78)	−3%	5,320	0.73 (0.68, 0.79)	3%
*Socio-economic group*								
Missing	50,322	0.04 (0.03, 0.04)	51,746	0.03 (0.02, 0.04)	−1%	50,546	0.04 (0.03, 0.04)	0%
Not deprived	89,966	(reference)	92,727	(reference)		90,631	(reference)	
Deprived	34,563	1.17 (1.14, 1.21)	35,749	1.16 (1.12, 1.19)	9%	34,884	1.17 (1.13, 1.20)	2%
No fixed abode	782	0.58 (0.46, 0.74)	1,031	0.40 (0.28, 0.56)	−70%	787	0.60 (0.47, 0.77)	7%
Foreign	140	0.42 (0.20, 0.86)	142	0.42 (0.20, 0.87)	2%	142	0.47 (0.23, 0.95)	14%
*Data year*								
1998 to 2003	49,456	(reference)	53,463	(reference)		49,895	(reference)	
2004 to 2009	50,348	1.83 (1.77, 1.91)	51,429	1.92 (1.85, 1.99)	−7%	50,751	1.82 (1.76, 1.89)	1%
2010 to 2015	75,969	2.08 (2.01, 2.16)	76,503	2.2 (2.12, 2.28)	−7%	76,344	2.08 (2.00, 2.16)	0%

a This refers to the percentage by which the log odds coefficient in each model is over-or under-estimated, compared to the reference standard model 100*[(logit_reference_–logit_comparison_/logit_reference_)], shown where the subgroup has a significantly increased risk of readmission in one year

b Models exclude records were sex is missing (n = 334 after deterministic match, 203 after probabilistic match and 176 for reference standard)

**Table 5:** False and missed matches after different thresholds for probabilistic matching

*Overall (1998 to 2015)*	Relaxed	Middle (as in main results)	Strict
False matches	1.8%	1.8%	1.4%
Missed matches	0.6%	0.7%	1.6%
*1998 to 2003*			
False matches	3.7%	3.6%	2.5%
Missed matches	2.3%	2.4%	6.8%
*2004 to 2009*			
False matches	1.2%	1.1%	1.2%
Missed matches	0.4%	0.4%	0.8%
*2010 to 2015*			
False matches	0.4%	0.4%	0.4%
Missed matches	0.1%	0.1%	0.2%

**Table 6:** Missing data on nhs number or postcode by data year

		1998–2003 (n = 99,200)			2004–2009 (n = 128,666)			2010–2015 (n = 189,373)	

	n	NHS number (%)	Postcode (%)	n	NHS number (%)	Postcode (%)	n	NHS number (%)	Postcode (%)
% missing									
*Age*									
0–3	68,225	61.2	19.0	77,270	6.5	41.3	89,896	0.7	47.3
4–7	17,774	17.5	2.6	13,112	4.5	2.9	13,435	1.7	2.8
8–11	13,221	10.8	4.0	11,833	4.4	4.1	10,309	1.2	2.0
12–15				15,370	4.7	4.0	14,144	1.5	3.7
16–19				11,081	3.4	3.8	24,304	1.9	4.0
20–23							37,285	1.8	3.9
Sex									
Missing	278	46.8	10.4	27	59.3	37.0	45	6.7	24.4
Male	43,996	48.7	15.2	60,181	5.9	27.3	99,675	1.1	22.8
Female	54,946	45.0	13.1	68,458	5.3	25.4	89,653	1.4	26.0
*Ethnic group*									
Missing	51,188	52.9	16.2	26,669	7.9	36.4	17,133	3.1	21.0
White	40,263	37.8	12.0	83,078	4.6	22.4	137,015	0.8	23.5
Mixed	142	16.9	2.8	2,698	5.5	27.2	6,078	1.2	34.0
Asian	3,399	42.1	8.6	8,812	6.0	28.2	15,461	1.5	29.8
Black	1,807	55.0	6.9	4,804	7.9	31.1	8,029	1.9	26.3
Chinese/Other	2,421	62.4	17.1	2,605	10.1	30.2	5,657	3.8	25.8
*Socio-economic group*									
Missing	13,937	65.7		33,808	6.7		46,042	0.7	
Low deprivation	59,914	43.5		67,485	4.8		100,721	1.0	
High deprivation	25,122	43.2		26,851	5.0		41,600	1.3	
Foreign	236	80.9		401	81.0		862	53.8	
No fixed abode	11	45.5		121	20.7		148	12.8	
